# Latent autoimmune diabetes in youth

**DOI:** 10.3389/fimmu.2025.1691377

**Published:** 2025-11-21

**Authors:** Qingxia Sun, Min Yang, Yiheng Jing, Lingzhe Meng, Chang Zhang, Jie Ruan, Na Wu

**Affiliations:** 1 Student Affairs Department, China Medical University, Shenyang, China; 2 Department of Pediatrics, Shengjing Hospital of China Medical University, Shenyang, China

**Keywords:** latent autoimmune diabetes in youth, latent autoimmune diabetes, slowly progressive insulin-dependent (type 1) diabetes mellitus, β-cell autoimmunity, type 1.5 diabetes

## Abstract

Latent autoimmune diabetes in youth (LADY) is an emerging and under-recognized subtype of autoimmune diabetes, occurring in young individuals who present with clinical features overlapping both type 1 and type 2 diabetes. LADY shares pathophysiological characteristics with latent autoimmune diabetes in adults (LADA) yet differs in its earlier onset and stronger autoimmune response. Due to its heterogeneous presentation, LADY is frequently misclassified as type 2 diabetes in youth, leading to delays in appropriate treatment. Moreover, there are currently no universally accepted diagnostic criteria or screening strategies specific to LADY. As a result, clinical management remains inconsistent and controversial. Given the rising incidence of autoimmune diabetes in youth and the clinical consequences of delayed diagnosis, there is an urgent need to better characterize LADY and develop age-specific diagnostic and therapeutic approaches. In this review, we summarize the current knowledge regarding the epidemiology, pathogenesis, clinical features, diagnostic challenges, and treatment strategies of LADY. We also highlight the existing knowledge gaps and propose directions for future research and clinical practice with the aim of improving the recognition and management of this unique diabetes subtype in the pediatric population.

## 1 Introduction

The global incidence of diabetes in children and adolescents is continuously rising (1, 2). Type 1 diabetes mellitus (T1DM), a disease with distinct potential etiological and pathological mechanisms, exhibits diverse clinical phenotypes, HLA associations, and autoantibody profiles (3). Latent autoimmune diabetes in adults (LADA), the most common form of autoimmune diabetes in adults, has received considerable attention (4, 5). The term “latent” distinguishes these slowly progressive cases from classic adult-onset T1DM (6). In 2019, the World Health Organization reclassified this condition as “slowly evolving, immune-mediated diabetes of adults” and categorized it under hybrid forms of diabetes (7). A similar subtype, termed latent autoimmune diabetes in youth (LADY) has been reported in children and adolescents (8, 9). However, research on LADY remains limited, its diagnosis is often overlooked by clinicians, and it remains a gray area in the spectrum of autoimmune diabetes.

**Figure 1:**
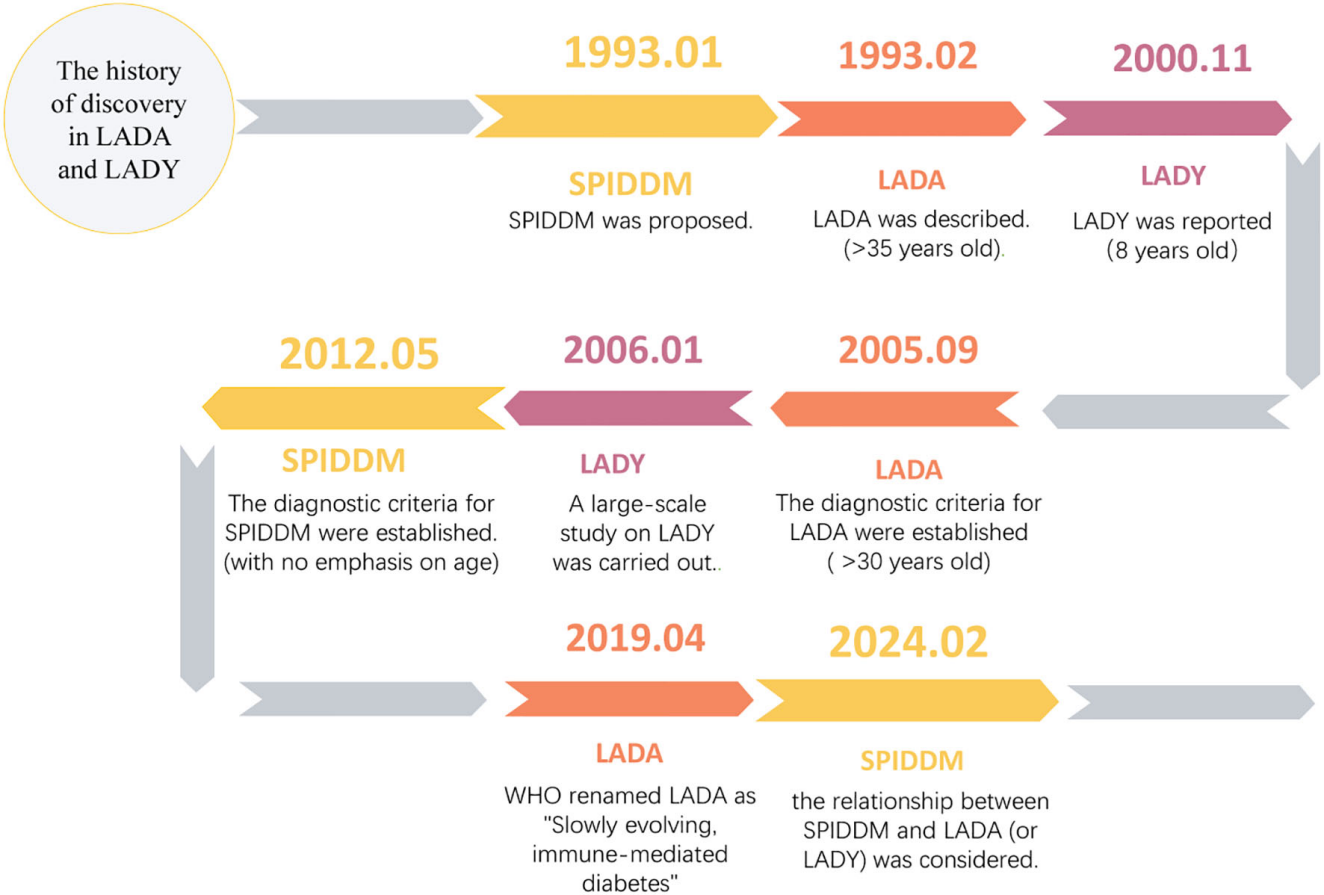
The history of discovery in LADA and LADY. SPIDDM, Slowly progressive insulin-dependent diabetes mellitus.

LADY was first proposed by Lohmann et al. in 2000 to describe children or adolescents exhibiting a T2DM phenotype but with β-cell autoimmunity (9). Clinically, LADY should be considered in young diabetic patients who lack classic T1DM features, are suspected of having type 2 diabetes mellitus (T2DM), but test positive for pancreatic autoantibodies—thus representing an intermediate type. Current literature reports the age distribution of LADY as 8 to 29 years (8–11). To date, the definition of LADY remains unclear (12). Although the cutoff age for distinguishing LADA from LADY varies across countries and regions, LADY is essentially equivalent to LADA with an earlier onset (**Figure 1**).

The classification of LADY has evolved with changing systems, but its core pathological mechanism consistently involves immune-mediated β-cell destruction. In the classic four-type classification, LADY is a special form of T1DM (based on T1DM criteria: “immune-mediated, characterized by presence of one or more autoimmune markers” and “β-cell destruction, usually leading to absolute insulin deficiency”) (13). Similarly, the Committee on Type 1 Diabetes of the Japan Diabetes Society uses the concept of slowly progressive insulin-dependent diabetes mellitus (SPIDDM) to describe LADA and LADY, still classifying them as T1DM (14). In 2019, the World Health Organization further refined diabetes classification into six categories: T1DM, T2DM, hybrid forms of diabetes, other specific types, unclassified diabetes, and hyperglycemia first detected during pregnancy (7). Both LADY and LADA fall under “slowly evolving, immune-mediated diabetes” within the hybrid forms.

Notably, the prevalence of LADY in young adults appears to be higher than that in adults (15). Studies have also suggested that LADY exhibits stronger autoimmunity than LADA (10). These findings indicated that LADY may be the predominant form of autoimmune diabetes in children and adolescents. Although the cutoff age between LADY and LADA lacks a universal standard (12), patients with LADY develop the disease early and live with it for decades. Without timely identification and treatment, the risk of complications and disease progression increases significantly. Furthermore, owing to an initial period of insulin independence, LADY is at risk of being misdiagnosed as T2DM (11). This underscores the urgent need to enhance awareness of LADY, elucidate its pathogenic mechanisms, and identify effective treatment strategies to mitigate its impact.

In this review, we summarize the reported onset and complex natural history of LADY, explore potential mechanisms underlying its highly autoimmune clinical phenotype, and highlight at-risk populations. We also compared the clinical characteristics of young LADY patients with those of other T1DM subtypes and adult patients with LADA. Finally, we discuss current management strategies and the challenges of implementing them in this population.

## 2 Epidemiology

Understanding the epidemiology of LADY is crucial for developing effective screening strategies. Similar to LADA, LADY patients initially experience a period of insulin independence, often leading to misclassification as T2DM. The UK Prospective Diabetes Study estimated that, regardless of age, 10-15% of patients with a presumptive diagnosis of T2DM had underlying autoimmune diabetes, with 58% requiring insulin within 6 years of diagnosis (16). LADY is not rare, but its proportion among patients diagnosed with T2DM varies widely in the literature, ranging from approximately 9% to 74% (17–21). The prominent US TODAY (Treatment Options for Type 2 Diabetes in Adolescents and Youth) study found that LADY accounted for 9.8% (118/1206) of obese children and youth diagnosed with T2DM (8). Another multicenter study involving 471 patients (from North and South America, Europe, Asia, and South Africa) reported that 10.2% of children and youth diagnosed with T2DM were actually LADY (22). Conversely, a smaller US study found a much higher proportion (74%, 14/19) of LADY among patients presenting with T2DM (17). Large multicenter studies have generally reported that approximately 10% of youth diagnosed with T2DM have LADY (8, 10, 15, 22). The significant variation in reported LADY proportions likely stems from differences in study design, participant inclusion criteria (e.g., age, sex, number and type of autoantibodies tested), sensitivity and specificity of antibody assays (e.g. RBA versus ELISA), and ethnicity or genetic background (5). Since the results of large-sample studies are similar, we believe that the difference in proportions is mainly caused by the small sample size. With the dramatic rise in obesity-associated T2DM (23, 24), the number of LADY cases may also increase substantially (10).

LADY shows no significant differences in terms of sex or geographic distribution (8, 10), but its ethnic distribution remains debated. Some studies have suggested that Caucasians have the highest probability of LADY, primarily due to higher antibody positivity rates (8). A study conducted in the United States in 2010 revealed that approximately 18.8% of screened Caucasian participants tested positive for antibodies, in contrast to less than 10% of screened Black (6.8%), Hispanic (8%), or American Indian (7.8%) participants (8). Conversely, a 2009 study indicated that among patients diagnosed with LADY, the representation of Caucasians, Hispanics, and Asians was nearly equivalent, whereas African American/Black patients constituted a comparatively small proportion (22).

Epidemiological data confirm that LADY is a significant entity, frequently misclassified as T2DM in youth populations. While reported prevalence varies widely, large multicenter studies consistently estimate that approximately 10% of youth clinically diagnosed with T2DM actually have LADY. This high rate of misdiagnosis underscores the critical need for improved screening strategies. Variation in prevalence estimates is largely attributable to methodological differences in study design, antibody testing protocols, and ethnic composition of cohorts. Crucially, LADY shows no significant sex or global geographic predilection. Alarmingly, the rising global tide of obesity-associated youth-onset T2DM suggests a parallel increase in LADY cases, demanding heightened clinical vigilance and standardized screening approaches.

## 3 Pathogenesis

**Figure 2:**
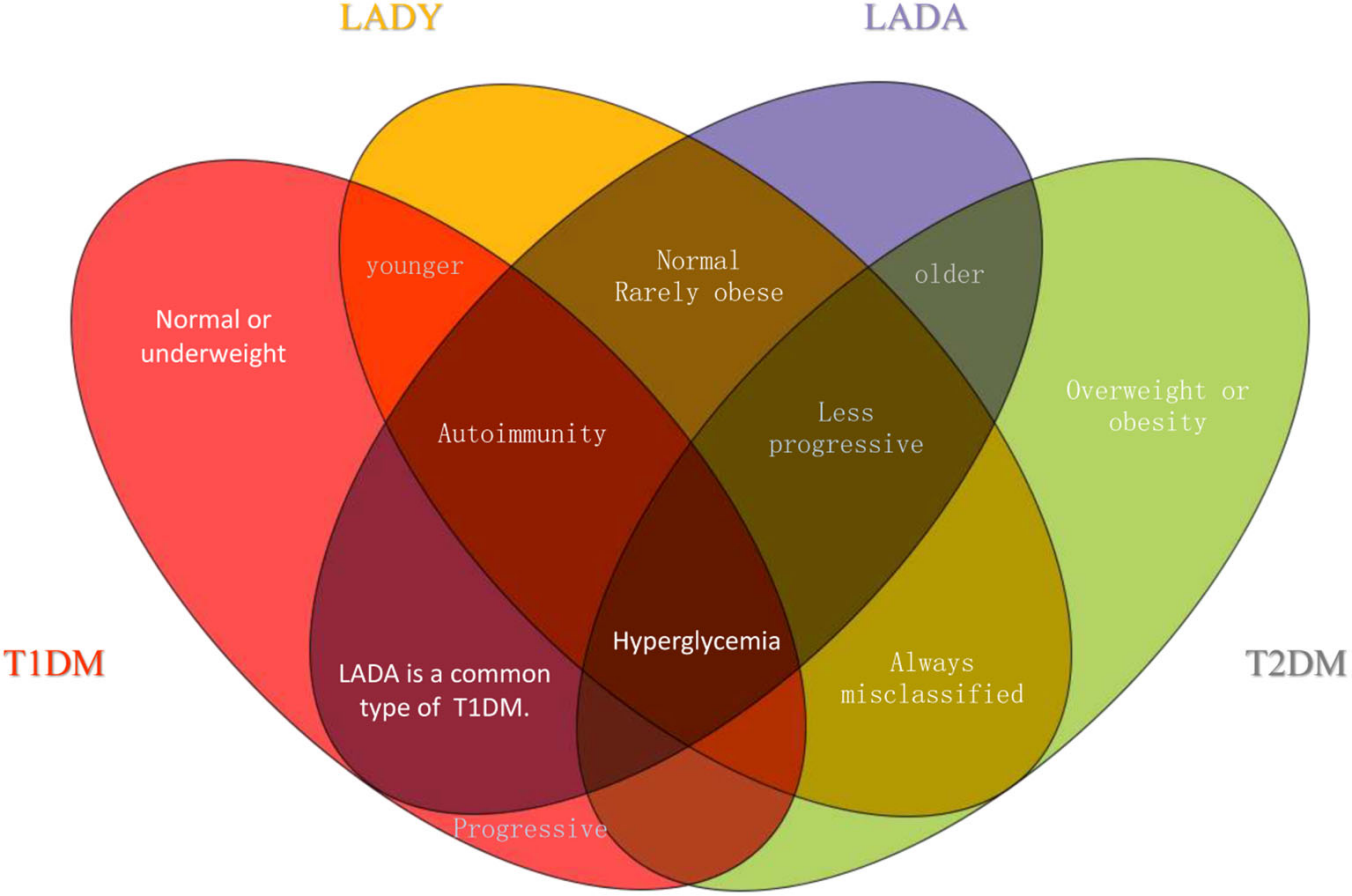
The venn diagram analysis show relationship between T1DM, T2DM, LADA and LADY. 1) T1DM is more progressive than other three types; 2) LADA is a common type of T1DM; 3)T1DM usually happens to the young, while T2DM usually happens to the old. LADY is similar to LADA, but happens to younger people; 4) T2DM is usually associated with obesity; 5) LADY is always misclassified as T2DM; and 6) All four types of diabetes present with symptoms of hyperglycemia.

Given the variation in reported prevalence of LADY among T2DM patients, understanding its underlying pathogenic mechanisms—including the observed higher ‘genetic load’ in early-onset cases—is crucial for accurate diagnosis and management. Compared with adult-onset autoimmune diabetes, young-onset disease carries a higher “genetic load” and is characterized by a greater number of diabetes-related autoantibodies (25). These genetic and autoimmune features correspond to more severe deterioration of β-cell function than seen in adult-onset cases. LADY exhibits stronger autoimmunity than LADA but does not initially require insulin, positioning it as an intermediate form between T1DM and LADA within the autoimmune diabetes spectrum (26) (**Figure 2**).

### 3.1 Genetics

Research on the genetics of LADY remains limited. Preliminary investigations into LADA genetics indicate that it may occupy a genetic nexus between T1DM and T2DM (27). Specifically, LADA appears to be significantly associated with major histocompatibility complex (MHC) loci characteristic of T1DM, as well as the TCF7L2 gene linked to T2DM (28). Furthermore, the risk conferred by HLA genotypes for LADA appears substantially higher than that for T2DM-associated genetic variants (29). A genome-wide association study (GWAS) involving 2634 LADA patients confirmed that its genetic basis primarily resembles T1DM but also includes variants associated with T2DM (30). A study on family history indicated that a family history of T1DM increases the risk of LADA six-fold, whereas a family history of T2DM doubles it (31). It is hypothesized that LADY may share similar genetic predispositions with LADA, but this requires confirmation in larger-scale studies.

LADY is likely to exhibit genetic characteristics similar to T1DM, including a significant association with HLA, linkage to DQA and DQB genes, and modulation by DRB genes. These HLA-DR/DQ alleles may act as either susceptibility or protective factors for LADY (32). In general, individuals with LADY show a higher prevalence of high-risk gene frequencies than adults, whereas protective genotypes are less common. Several studies support this conclusion: pediatric patients have a higher prevalence of the high-risk HLA-DQB1*02/*0302 genotype than adults (25). Furthermore, the frequencies of the DR3-specific susceptibility haplotype, overall susceptibility haplotypes, and high-risk genotypes are more pronounced in classic T1DM and LADY patients than in those with LADA or T2DM (26). After controlling for variables such as age, sex, and body mass index (BMI), the prevalence of high HLA genetic risk genotypes (DR3/3, -3/9, and -9/9) in LADY patients is comparable to that in T1DM patients, both elevated (The total susceptible HLA haplotypes included DR3, DR4-DQB1*0401, DR4-DQB1*0302, and DR9) (33). Conversely, protective genotypes are relatively rare (25): the prevalence of low or no genetic risk genotypes (DRX/X) is lower among T1DM and LADY patients than LADA or T2DM patients (26). In conclusion, the distribution of HLA susceptibility genes in patients with T1DM and LADY is more similar, whereas LADA is closer to T2DM. The frequency of high-risk HLA susceptibility genotypes and total susceptible haplotypes is higher in T1DM and LADY than in LADA and T2DM, while low-risk or non-risk HLA genotypes are less common in T1DM and LADY compared to LADA and T2DM. Moreover, mutations activating STAT3 have been implicated in the onset of early-onset autoimmune diseases (34).

LADY may also exhibit genetic traits associated with T2DM, though research on T2DM-related genetic factors in LADY is scarce. Previous studies suggest LADA may be linked to T2DM pathogenesis: LADA involves insulin deficiency due to autoimmune β-cell destruction and, to some extent, insulin resistance (35). A UK study revealed that 33% of individuals with LADA had family members diagnosed with T2DM (36). Beyond TCF7L2, the Progress in Diabetes Genetics in Youth (ProDiGY) study—analyzing data from diverse cohorts in the TODAY, SEARCH, and T2D-GENES studies—identified six genome-wide significant loci associated with T2DM: MC4R, CDC123, KCNQ1, IGF2BP2, and SLC16A11, located near or within PHF2 (37). Given the T2DM-like phenotype observed in LADY, future investigations should explore the potential associations between these genes and LADY.

Notably, existing genetic studies on LADY have focused on T1DM- or T2DM-related genes, assuming LADY shares susceptibility alleles with these diseases. This has limited the discovery of LADY-specific genes. Moreover, most studies have been conducted in a single population. Larger and more diverse studies are required to clarify the genetic architecture of LADY.

### 3.2 Immunity

Existing research on the pathogenesis of LADY is limited, but numerous studies have indicated that LADA is primarily caused by cell-mediated immunity (38, 39), suggesting LADY may also be cell-mediated. Given that HLA genes encode MHC molecules with crucial immune regulatory functions, immune dysregulation in LADY is unsurprising. Genetics can influence disease onset through antibodies: logistic regression analysis suggests HLA susceptibility haplotypes are a risk factor for multi-epitope positivity for glutamic acid decarboxylase antibodies (GADA) in patients with autoimmune diabetes (12). Anti-tyrosine phosphatase antibody (IA-2A) positivity in both children and adults is associated with the DQB1*0302/x genotype (25).

It is well-established that autoreactive CD8^+^ T cells, upon recognizing antigenic determinants expressed on islet β-cell surfaces in association with MHC-I molecules, exert cytotoxic effects via degranulation and perforin release (40). Pancreatic biopsies revealed that CD8 ^+^ T cells are a major component of the insulitis immune cell infiltrate in patients with LADA (41). Although the total number of immune cells infiltrating islets shows no significant difference between T1DM and LADA patients(or their respective rat models), the composition differs: CD8^+^ T cell levels are lower in LADA than in T1DM (42). This phenomenon may relate to less severe islet destruction in LADA. Additionally, regulatory B cells (Bregs)—which modulate T cell responses and suppress inflammation by secreting cytokines such as IL-10 and IL-35—are less frequent in LADA patients than in T1DM patients (43, 44), further explaining the slower β-cell destruction in LADA. Reduced numbers and functional defects in regulatory T cells (Tregs) are also considered key contributors to autoimmunity in LADA (45). A better understanding of T-cell and B-cell interactions will provide new insights into LADA pathogenesis, potentially applicable to LADY.

Increasing evidence links innate immunity to the pathogenesis of autoimmune diabetes (46). Studies found that peripheral blood mononuclear cells (PBMCs) from LADA patients can suppress insulin secretion from human islets, indicating cell-mediated autoimmune responses (39).

Islet autoantibodies are generally considered an epiphenomenon rather than a key pathogenic factor in islet cell destruction, but they are widely used to distinguish autoimmune from non-autoimmune diabetes and can indicate the strength of autoimmunity (5). Intramolecular Ab epitope spreading is a phenomenon in which new epitopes within the same molecule are recognized over time, and can be associated with progression to disease (47). Schlosser et al. proposed that in susceptible individuals, the autoimmune response may undergo intramolecular epitope spreading from the NH_2_-terminal epitope of the GAD65 protein (GAD65-N) to the middle region epitope of the GAD65 protein(GAD65-M) (47). GAD65 antibody epitope specificity testing showed that the frequencies of COOH-terminal epitope of the GAD65 protein (GAD65-C) Ab and GAD65-MAb were higher in the LADY and T1DM groups than in the LADA group, whereas GAD65-NAb frequency was higher in the LADA group than in the juvenile T1DM group (26). In terms of the number of GADA epitope types (GAD65-Nab, GAD65-Mab and GAD65-Cab), the positivity rate for GADA with two or more epitopes was higher in patients with T1DM and LADY, with no statistically significant difference between the two groups. However, the rate in LADA patients was significantly lower than in those with T1DM and LADY, suggesting that the humoral immune response in T1DM and LADY patients is stronger and more extensive (26). Compared with LADA, LADY’s GAD65 epitope characteristics are closer to those of T1DM. When grouped by age, latent autoimmune diabetes forms a continuous age-related spectrum from LADY to LADA, with decreasing GADA titers, reflecting stronger autoimmunity in LADY (10, 26). A study comparing islet antigen-specific cellular immune responses in children and adults with newly diagnosed T1DM found that pro-inflammatory autoreactivity was more prevalent, targeted to a broader range, and was more focused on insulin/proinsulin in children—consistent with stronger autoimmune responses in the younger individuals (48).

Gut microbiota plays a critical role in postnatal immune system development. Numerous microorganisms and their metabolites have immunomodulatory properties that significantly influence immune development and function. Increasing evidence links gut microbiota dysbiosis to the onset of autoimmune diabetes. A comprehensive multi-omics investigation has identified associations among gut microbiota, fecal metabolites, serum metabolites, and clinical phenotypes (including islet autoantibodies, glucose metabolism, islet function, and inflammatory cytokines) in LADA patients (49).

### 3.3 Others

Autoimmune diabetes is a chronic disease resulting from the interplay of multiple factors. Beyond genetics, triggers of autoimmunity remain unclear. Psychological stress is associated with T1DM development in children (50) but not observed in LADA (51). The Swedish ESTRID study indicated fatty fish consumption is associated with a reduced risk of LADA (52), consistent with findings that it lowers T1DM risk in children (53–55). This effect may stem from the anti-inflammatory and immunomodulatory properties of omega-3 fatty acids, abundant in fatty fish. Erythrocyte fatty acids in children are also associated with islet autoimmunity risk: higher levels of eicosapentaenoic acid (EPA), n-3 polyunsaturated fatty acids (PUFAs) in infancy, and conjugated linoleic acid (CLA) after infancy are linked to lower risk, whereas higher levels of certain even-chain saturated fatty acids (SFAs) and monounsaturated fatty acids (MUFAs) increase risk (56).

The comparatively slower decline in β-cell function in LADY, relative to classical type 1 diabetes mellitus (T1DM), likely reflects the combined influence of genetic, immunological, and metabolic factors. Distinct HLA-DR/DQ haplotypes and GAD65 epitope profiles identified in LADY suggest that age-dependent genetic and antigenic features shape disease susceptibility and modulate the pace of β-cell loss (12, 57). Immunologically, LADY appears to represent the younger extreme of an autoimmune diabetes spectrum extending to LADA—marked by stronger islet autoimmunity than in adults but a less aggressive immune assault than in childhood-onset T1DM (57). In youth-onset autoimmune diabetes, greater autoantibody load has consistently been associated with faster β-cell decline (58), whereas obesity-related inflammation and insulin resistance may further modify disease progression (59, 60). Collectively, these observations indicate that LADY occupies a distinct immunometabolic niche—defined by genetic background, developmental stage, and inflammatory environment—that contributes to its more gradual β-cell deterioration.

Understanding these interrelated mechanisms also emphasizes the diagnostic challenges inherent in differentiating LADY from classical T1DM or T2DM and underscores the need for standardized, age-specific diagnostic frameworks.

## 4 Screening and diagnosis

### 4.1 Screening

No dedicated screening studies for LADY exist, but tools developed for LADA may offer insights. Given that both LADA and LADY have insidious onset and are difficult to distinguish from T2DM, defining clinical features to identify hyperglycemic patients at risk of latent autoimmune diabetes could significantly facilitate early detection.

In current clinical practice, LADA is often considered for lean patients not requiring insulin therapy. Although a low BMI is common in LADA and LADY patients, those with a high BMI are still at risk. A clinical risk score developed by the Fourlanos team through a retrospective study (Including LADA and T2DM patients aged 30–75 years) showed that meeting any two of the following criteria yielded 90% sensitivity and 71% specificity for identifying LADA: (1) age of onset < 50 years, (2) acute symptoms (polydipsia/polyuria/significant weight loss), and (3) BMI < 25 kg/m² (61). Patients failing to meet two criteria could be ruled out for LADA. The LADA criteria proposed by Monge et al. required suspected T2DM patients over 50 years old to simultaneously fulfill: (1) normal or low BMI, (2) fasting blood glucose≥15 mmol/l and/or HbA 1c≥10% despite adequate compliance to diet and treatment, and (3) decreasing body weight≥10% in the previous 3 months despite a constant diet. This tool identified three-quarters of LADA patients (62). Both studies suggest that hyperglycemia with normal/low BMI is a significant warning sign for LADA. However, it is important to note that some studies confirmed LADA diagnosis in patients with BMI >25 kg/m² (63, 64)and reported LADY cases can have BMIs up to 28 kg/m² (9). Therefore, a BMI >25 kg/m² cannot be used to exclude LADA or LADY.

Serum C-peptide levels are easier to measure than autoantibody levels. Since most patients meeting the LADA criteria also have low C-peptide levels, one study enrolled LADA patients based on low serum C-peptide (<0.8 ng/mL) (participants diagnosed within the past 6 months, age≥25 years, baseline HbA1c 6.5%) (65). While the sensitivity and specificity of this criterion require further investigation, it provides a rationale for LADY screening. Wang et al. developed and validated a prevalence model for LADA among people initially diagnosed with T2DM (≥18 years) (66). Key predictors included age, ketosis, smoking history, 1-hour glucose, and 2-hour C-peptide levels. A multivariate logistic regression model based on these factors showed high sensitivity (78.57%) and specificity (67.96%) for LADA diagnosis (66).

Additionally, a history of autoimmune diseases and family history of diabetes may suggest LADA (61). Studies show that individuals with a family history of diabetes have a fourfold increased prevalence of LADA (67), and prospective data indicate that over 11 years of follow-up, subjects with siblings with diabetes had a 2.5 times higher risk of developing LADA than those without (67). Glycoprotein phospholipase D (GPLD1) may also be a potential candidate plasma protein for early differentiation between LADA and T2DM (68).

Based on these data, we propose considering LADY in patients meeting the following criteria: (1) does not require insulin for glycemic control, (2) present in youth (age <29 years), (3) personal/family history of autoimmune diseases or diabetes (not specific to LADY); (4) acute symptoms of polydipsia, polyuria, or weight loss; and (5) low serum C-peptide (<0.8 ng/mL). Importantly, BMI >25 kg/m² should not be used as an exclusion criterion for LADY.

### 4.2 Diagnosis

Islet autoantibodies are biomarkers of β-cell autoimmunity that distinguish LADA/LADY from T2DM (4, 20), though rare antibody-positive “T2DM” cases have been reported (16). Any antibody-positive patient should be carefully evaluated.

The antibodies used to diagnose LADY include GADA, IA-2A, cytoplasmic islet cell autoantibody (ICA), zinc transporter 8 autoantibody (ZnT8A), and insulin autoantibody (IAA). Typically, 85-90% of individuals have multiple autoantibodies present when hyperglycemia is first detected (69), with GADA being the most prominent. Autoantibody positivity rates vary among populations, and measuring only one antibody may underestimate LADY prevalence. In a multicenter study of 1803 subjects aged < 30 years, GADA, IA-2A, and ZnT8A positivity was found in 88.1% (119/135), 30.4% (41/135), and 28.1% (38/135) of LADY patients, respectively (10). Autoantibodies in patients can fluctuate (70). In such cases, regular follow-up to dynamically observe disease changes is necessary.

β-cell autoantibodies can help differentiate between disease types and progression. In adults, antibody type can distinguish acute-onset T1DM from LADA, as GADA and ICA indicate slow progression, while IA-2 presence is associated with an acute-onset clinical phenotype. Early research suggested that in children and adolescents, all antibody types can be detected in both T1DM and LADY patients, making the antibody type seemingly unable to distinguish between these two diseases (20). Clinicians should also be vigilant for patients who are antibody-negative at initial diagnosis but seroconvert to antibody-positive during follow-up. Cases have been reported where IA-2 antibodies appeared three years after disease onset (70). Children with LADY show no differences from those with T2DM in terms of age, sex, insulin resistance, and glucose metabolism. The two groups can only be distinguished by measuring β-cell antibodies (20).

Owing to the slower rate of β-cell loss in LADA, the period of insulin independence after onset distinguishes LADA patients from classic adult-onset T1DM patients (who require insulin within 3 months of diagnosis).

Referring to the diagnostic framework of LADA (7), LADY has often been summarized by three criteria: (1) positive pancreatic autoantibodies (especially GADA positivity, but LADY cannot be excluded if GADA is negative but other antibodies are positive), (2) onset during youth (8–29 years), and (3) no requirement for insulin therapy for at least 6–12 months after diagnosis. However, applying adult-derived diagnostic criteria to pediatric populations may not be fully appropriate, as developmental, immunological, and metabolic characteristics differ substantially between youth and adults. Therefore, there is an urgent need to develop evidence-based, youth-specific diagnostic algorithms for LADY. Such algorithms should consider whether universal islet autoantibody screening is warranted in newly diagnosed adolescents and whether age-adjusted thresholds for antibody titers are required to enhance diagnostic accuracy.

Although the cutoff age between LADA and LADY varies across countries and regions (26), LADY essentially represents LADA occurring at a younger age. For example, Welters et al.’s large study included patients under 18 years of age for LADY research (71), while Xiang et al.’s LADY study included patients aged 15–29 years (33). Lohmann et al. used an age >35 years criterion for LADA patients (64), a standard proposed by WHO/ADA in 1997 (72). In 2005, the Immunology of Diabetes Society proposed three main diagnostic criteria for LADA: (1) adult-onset age >30 years, (2) presence of circulating islet autoantibodies, and (3) no insulin requirement for at least 6 months after diagnosis(4). Considering these differing opinions and their publication dates, the use of 30 years as a cutoff age for distinguishing LADY and LADA warrants consideration.

## 5 Clinical features

LADY onset characteristics are similar to LADA, involving gradual autoimmune destruction of pancreatic β cells, leading to reduced insulin production, subtle symptoms, and slow progression (9). LADY patients exhibit clinical and metabolic features intermediate between T1DM and T2DM (73). They test positive for islet cell antibodies but do not require insulin therapy at diagnosis, and their cytokine levels shows no significant difference from those of classic T1DM patients (33). Patients may experience polydipsia, polyuria, and weight loss, but these are less pronounced than those in classic T1DM (11). Some patients present with ketonuria and elevated blood glucose (74), and may develop diabetic ketoacidosis (DKA), leading to long-term complications. Early clinical presentation resembles that of T2DM. However, compared to T2DM patients, LADY patients have less obesity, hypertension, dyslipidemia, insulin resistance, lower fasting C-peptide (FCP) levels, and higher adiponectin levels (10, 33). Consistent with these findings, further research indicates that the clinical manifestations of LADY lie between those of T1DM and T2DM: BMI and hs-CRP progressively increase from T1DM to LADY to T2DM, while adiponectin progressively decreases (75). Compared to younger patients, older patients have a higher prevalence of metabolic syndrome and higher levels of β-cell function markers [FCP, postprandial C-peptide (PCP), and HOMA2-β (an index in the HOMA2 model for assessing beta-cell function)] (10) (**Table 1**).

**Table 1:** Comparison of LADY, LADA, classic T1DM and T2DM.

	Classic T1DM	LADY	LADA	T2DM	Reference
Proportion of diabetes mellitus	10-15%	About 10% of adolescence	3.79%-4%	85-90%	(7)
Age of onset	Most common in children or adolescents	<18 years / <29 years	Usually >30–35 years	Common in middle-aged and elderly people	(4, 10, 33, 64, 71)
Progressive	Rapidly	Slowly	Slowly	Hidden onset of disease	(7)
HLA susceptibility	Increased	Increased	Higher	No change	(26)
Gene	susceptible HLA haplotypes were DR3, DR4, DRB1*0405-DQA1*0301DQB1*0302,and DR9	Related to genes associated with T1DM and T2DM	Related to genes associated with T1DM and T2DM	TCF7L2 etc.	(25, 26, 37)
Body mass index	Normal or underweight	Normal Rarely obese	Normal Rarely obese	Overweight or obese	(61, 61)
Clinical manifestations	"Three more and one less", prone to ketoacidosis	Features of both T1DM and T2DM	Features of both T1DM and T2DM	"Three more and one less", most often accompanied by metabolic syndrome	(9–11, 33, 73)
Autoimmunity	Increased	Greater than LADA	Slightly raised	Absent	(26)
Total antibody detection rate(%)	96-100	83.6-95.3	Antibody positivity is one of the diagnostic criteria for LADA	9.8-73.7	(8, 17, 35, 71)
GADA(%)	40.7-66	55.5-88.1	67-100	6.3-36.8	(8, 17, 20, 35, 71)
IA-2A(%)	30.2-66	30.4-57.1	11-40	7.4-42.1	(8, 17, 20, 35, 64, 71)
ICA(%)	14.5-52	60.9	69-76	4-52.9	(16, 17, 20, 64, 71)
IAA(%)	25.8-53	71.8	14.6-41	26.3-40	(17, 20, 71)
Zn-T8(%)	29-32.2	28.1-66.7	21.4-27	NA	(71)
Insulin dependence	At onset	>6 months (even years) after onset	>6 months (even years) after onset	Years after onset	(7)

Some reports suggest that Maturity-Onset Diabetes of the Young (MODY) can coexist with autoimmune diabetes. Regarding antibody frequency in MODY patients, McDonald et al. reported frequencies similar to controls (76), while others reported higher frequencies (17% and 25%, respectively) (77, 78). Currently, only one case report has described the coexistence of MODY and LADY, and a 12-year-old Japanese girl had both MODY1 and LADY. Initially, glycosuria, elevated fasting glucose (11.6 mmol/L), and HbA1c (10.5%) levels were detected, with no β-cell-related autoantibodies. Based on molecular analysis, the patient was diagnosed with MODY1 (70). Her parents did not have diabetes, but her uncle had T2DM. Her blood glucose was initially well-controlled with insulin but occasional hypoglycemia led to a switch to oral hypoglycemics (alogliptin and glimepiride) (70). Three years later, she tested positive for IA-2 antibodies. Owing to poor glycemic control, the treatment was changed to liraglutide. Throughout the study, IA-2 antibodies remained positive in the radioimmunoassay and enzyme-linked immunosorbent assay. Based on the clinical course, she was diagnosed with late-onset autoimmune diabetes, LADY (70).

Pancreatic disease is an extremely rare cause of diabetes. Although alcohol abuse is the primary cause of chronic pancreatitis worldwide, a peculiar form of chronic pancreatic insufficiency and diabetes exists in certain tropical regions of the world, not related to harmful use of alcohol. These conditions are referred to as tropical chronic pancreatitis (TCP) and fibrocalculous pancreatic diabetes (FCPD) (79). FCPD can coexist with LADY as well. A case of FCPD was reported for which antibody and cellular immune characteristics were determined (80). The patient, a 27-year-old Thai woman, had ICA that were detected by both immune-peroxidase staining and an indirect ELISA. This suggested an association between autoimmunity and FCPD. Based on the information above, we can infer that she has combined FCPD and LADY.

Notably, as an autoimmune disease, the onset of LADY is closely associated with other autoimmune disorders, including thyroid autoimmunity, celiac disease, vitiligo, adrenal insufficiency and others (81–84). The link between diabetes and thyroid autoimmunity has been well established. Autoimmune thyroid disease (AITD) is the most common autoimmune disease coexisting with diabetes, particularly autoimmune diabetes (85, 86). AITD is characterized by the presence of thyroid peroxidase autoantibodies (TPOA) and thyroglobulin autoantibodies (TGA); similarly, higher TPOA or TGA titers correlate with a higher AITD risk (87). The serum positivity rate for TPOA is higher in LADY patients than in LADA patients (81), and LADY patients with high GADA titers have a higher frequency of thyroid autoantibodies than those with low GADA titers. Further analysis revealed that high GADA titers correlated positively with thyroid autoantibody positivity only in male patients (81).

## 6 Treatment

**Figure 3:**
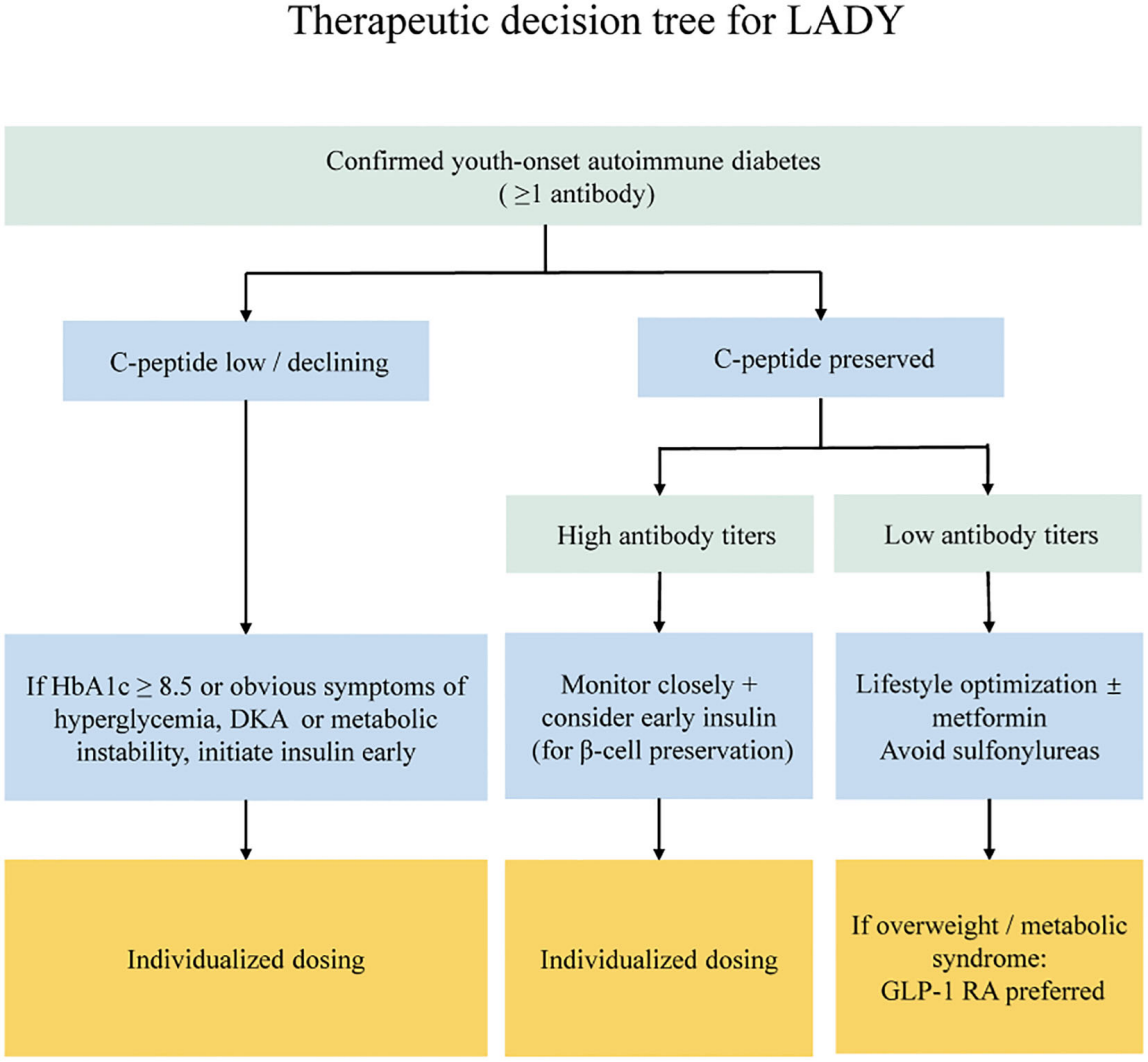
Suggested management algorithm for LADY based on C-peptide status, antibody strength, and metabolic profile. Left branch: patients with low or declining C-peptide should start early insulin therapy, with individualized dosing to preserve residual B-cell function. Right branch: patients with preserved C-peptide are stratified by antibody profile-high titers/multiple antibodies warrant close monitoring and consideration of early insulin, while low titers/single antibody positivity can be managed with lifestyle optimization + metformin, sulfonylureas should be avoided. In overweight patients or those with metabolic syndrome, GLP-1 receptor agonists are preferred.

Clinical management strategies for LADY remain controversial. While preliminary diagnosis and treatment consensus exists for LADA (88), unified treatment pathways for specific adolescent population are lacking. There are no existing relevant pediatric RCTs. Current clinical practice relies heavily on clinical experience and professional judgment, resulting in significant individualization. Importantly, accurate classification of young diabetic patients early in the disease course is often difficult. Thus, clinical guidelines recommend a staged management strategy that prioritizes glycemic control and management of related metabolic disturbances at diagnosis (regardless of the final classification) and adjusts treatment based on specific diagnostic markers such as islet autoantibodies once available (89) (**Figure 3**).

### 6.1 Pharmacological treatments

#### 6.1.1 Insulin therapy

Available literature indicates that patients with LADY have initial fasting blood glucose levels ranging from 7.0-11.8 mmol/L and HbA1c levels between 6.5%-10.5% (9, 11, 69–71). Clinically, most achieve glycemic targets early through lifestyle interventions (diet control combined with exercise) or oral hypoglycemic agents. Notably, even when autoantibody positivity is detected, clinicians often delay insulin initiation—likely due to the longer persistence of residual β-cell function in youth.

Evidence regarding optimal timing for insulin therapy initiation and dosage selection is limited. The potential benefits and drawbacks of initiating insulin therapy early in the disease course remain to be clarified. Whether insulin should be administered in the early stages of LADY or is optimal treatment regardless of disease stage remains undetermined. Existing studies on early insulin therapy for preserving β-cell function in patients with LADA are inconclusive. A randomized trial comparing insulin and sulfonylureas in LADA found that insulin was more effective in preventing β-cell failure (90). Research suggests sulfonylureas may accelerate β-cell exhaustion, whereas early insulin initiation could help preserve β-cell function (91). Conversely, some studies argue that early insulin treatment in LADA does not necessarily preserve β-cell function but primarily improves metabolic control (92). These conflicting findings highlight the need for further research to clarify optimal timing and strategies for insulin therapy in autoimmune diabetes subtypes such as LADA and LADY.

By current definitions, individuals with LADY generally remain insulin-independent for 6 to 12 months after diagnosis. Identifying patients with LADY early— before insulin dependence develops—is key to timely insulin initiation. A multicenter study involving 304 LADY patients (primarily from Germany and Australia) showed that 75.9% received insulin therapy, initiated on average 1.1 years after diagnosis, with the average dose gradually increasing from 0.5 IE/kg at start to 0.8 IE/kg at follow-up (71). Lohmann et al. reported an 8-year-old who started insulin 9 months after diagnosis (0.15 IU/kg/day), achieved HbA1c 6.5%, and had the dose reduced to 0.05 IU/kg/day three months later (9). This case suggests insulin requirements in young patients may be dynamic, but specific dose adjustment patterns require validation in larger studies.

#### 6.1.2 Oral hypoglycemic agents

LADY patients are difficult to identify, and some receive oral medications before definitive diagnosis. Large studies have shown that 23.4% of patients with LADY used oral hypoglycemic agents or glucagon-like peptide-1 receptor agonists (GLP-1 RAs) (71). Metformin, GLP-1 RAs, SGLT-2 inhibitors, DPP-4 inhibitors, and sulfonylureas were used in 22.7%, 1.8%, 1.1%, 1.1%, and 0.7% of patients, respectively (71).

The relatively high use of metformin among LADY patients may reflect the frequent coexistence of insulin resistance and the fact that metformin remains the only oral hypoglycemic agent approved for children and adolescents aged ≥10 years with type 2 diabetes mellitus (T2DM). In a few expert consensus statements addressing LADA treatment (93), metformin is often regarded as a potential adjunctive therapy rather than a clearly recommended first-line monotherapy. These statements consistently note that evidence supporting metformin monotherapy in LADA is limited and that it does not appear to delay the eventual progression to insulin dependence. From a molecular mechanism perspective, metformin may have both indirect and direct protective effects on pancreatic β-cells. Its classical mode of action involves inhibition of hepatic gluconeogenesis and improvement of peripheral insulin sensitivity, leading to reduced hyperglycemia and mitigation of glucotoxic stress on β-cells—thus providing indirect protection (94). In addition, experimental studies have demonstrated direct β-cell effects through activation of the AMPK signaling pathway, promoting autophagy-mediated clearance of damaged organelles and exerting antioxidant and anti-apoptotic actions (95). Nevertheless, despite these promising molecular insights, clinical evidence—particularly from trials in adolescents—has not demonstrated that metformin, whether used alone or in combination with insulin or other agents, effectively prevents or delays progressive β-cell decline in youth with T2DM or impaired glucose tolerance (96). Moreover, *in vitro* data indicate that metformin may inhibit the differentiation and functional maturation of human embryonic stem cells into pancreatic β-cells (97). Collectively, these findings suggest that the use of metformin in LADY remains exploratory. Further high-quality, evidence-based studies are required to clarify its therapeutic role. At present, metformin should not be regarded as a first-line therapy but rather as an adjunctive option in selected insulin-resistant or overweight patients.

Sulfonylureas are the only class with detailed pre- and post-treatment data in published LADY case reports (11, 69), used until loss of control necessitates insulin initiation and dependence. Importantly, patients were not diagnosed with LADY when sulfonylureas were prescribed.

Youth are often excluded from oral hypoglycemic clinical trials, so many LADY treatment recommendations are extrapolated from adult data rather than evidence proven in youth. Furthermore, due to limited efficacy and safety data in adolescents, some adult-approved drugs are not approved for youth, reducing options for achieving glycemic normalization in young-onset patients.

Sulfonylureas are not recommended for LADY treatment. Current research indicates they accelerate β-cell exhaustion in both LADY and LADA patients (69, 91, 98, 99), leading to declining C-peptide levels, persistent antibodies, and rapid progression to insulin dependence (100). A systematic review concluded no clear evidence that sulfonylureas are preferable to other forms of therapy for LADA (99). Rajkumar et al. explicitly stated that sulfonylureas were a poor choice for LADA (100). Guidelines also advise against using acarbose or sulfonylureas in combination with insulin for children and youth with T1DM, as they may increase the risk of hypoglycemia without improving glycemia (101).

GLP-1 RAs have garnered attention for their immunomodulatory effects, attributed to GLP-1 receptor expression on immune cells (102). Preclinical studies have highlighted their ability to reduce β-cell apoptosis, stimulate β-cell regeneration, and slow disease progression in T1DM mouse models (103). Clinical studies have also shown that dulaglutide improves β-cell function and reduces HbA1c levels in LADA patients (104). At a year post-diagnosis, dulaglutide treatment resulted in comparable HbA1c reductions in T2DM and LADA patients and seemed more effective in LADA patients with low autoantibody levels than in those with high levels (104). Research on GLP-1 RAs specifically targeting LADY remains limited. However, existing studies suggest that discontinuation of GLP-1 RA treatment is associated with rapid weight regain, implying that sustained administration may be essential for maintaining stable weight and metabolic function. Consequently, further investigation is warranted to establish evidence-based guidelines for GLP-1 RA use in the management of LADY.

Currently, dipeptidyl peptidase-4 (DPP-4) inhibitors are not employed in the treatment of LADY. However, emerging data suggest that the combination of DPP-4 inhibitors with insulin therapy may partially preserve pancreatic β-cell function in LADA compared to insulin monotherapy (105, 106). One study showed that sitagliptin altered T-cell subsets and phenotypes in LADA by increasing protective Th2 cells and reducing pathogenic Th17 cells, resulting in improved glycemic control in LADA patients after 12 months (107). Furthermore, a pilot study indicated that sitagliptin might sustain β-cell function in LADA patients for a duration of at least four years more effectively than insulin alone, potentially through immunomodulatory mechanisms (108). The safety of DPP-4 inhibitors in children has not been fully established. A clinical study administering sitagliptin 100 mg/day to young patients with T2DM aged 10–17 years showed good tolerability and safety similar to that in adults (109, 110), but they are not yet recommended in guidelines. Consequently, the application of DPP-4 inhibitors in LADY warrants comprehensive investigation.

SGLT-2 inhibitors show clinical potential for LADY due to their unique insulin-independent glucose-lowering mechanisms. Although research on SGLT-2 inhibitors in LADA patients is scarce and suggests inferior efficacy compared to DPP-4 inhibitors (65) (HbA1c reduction: DPP-4i 1.1 ± 0.3% *vs*. SGLT2i 0.8 ± 0.13% at 3 months), international multicenter randomized clinical trials involving over 5000 T1DM patients have confirmed the efficacy and safety of adding SGLT-2 inhibitors to existing insulin regimens (111–118). T1DM patients using SGLT-2 inhibitors must monitor for euglycemic diabetic ketoacidosis (euDKA), which often occurs with normal or mildly elevated blood glucose (119). For non-insulin users with low/moderate C-peptide levels, SGLT-2 inhibitors may mask biochemical markers of progressive insulin deficiency (e.g., postprandial hyperglycemia), so patients should monitor blood and urine ketones (119). Though approved for T2DM and some T1DM patients (particularly overweight individuals) (88), their efficacy and safety in LADY require further validation.

Notably, thiazolidinediones (TZDs), besides acting as PPARγ agonists to improve insulin resistance in T2DM treatment, are increasingly recognized for their unique anti-inflammatory properties and β-cell-protective effects (120–122). Animal studies have shown that they maintain islet structure, enhance β-cell stress resistance, and promote proliferation (121, 122). Clinical studies further support the potential application of TZDs in islet protection therapies (123, 124). A study of 54 LADA patients confirmed that after 18 months, the rosiglitazone group had higher PCP levels than the sulfonylurea group, and the rosiglitazone group achieved better HbA1c levels over 3 years (mean HbA1c 6.49%) than the SUs group (mean HbA1c 7.06%) (125). However, whether they can be applied to LADY still requires further research to confirm.

Overall, oral agents should prioritize glycemic control and β-cell preservation while avoiding autoimmunity accelerators (e.g., sulfonylureas). GLP-1 RAs and DPP-4 inhibitors are preferred based on immunomodulatory potential, but most data are extrapolated from LADA/adult studies, and LADY-specific trials are scarce.

#### 6.1.3 Immunomodulatory therapies

Immunotherapy represent a promising area for future research in LADY. Although no LADY immunotherapy has been reported, immunological intervention trials targeting LADA shown some effectiveness in maintaining C-peptide levels and in enhancing glycemic control (126, 127). Specifically, GAD65 vaccination in LADA patients showed good safety and no detrimental effect on islet β-cell function over a 5-year follow-up period (128). However, the tested molecules are not yet commercially available, and their potential effects on LADY are anticipated.

Teplizumab, a CD3-directed monoclonal antibody that preserves β-cell function in people with Stage 3 T1DM and delays the onset of Stage 3 T1DM in those with Stage 2 T1DM, represents the sole therapeutic agent to date that has received regulatory approval for the purpose of delaying the progression from Stage 2 to Stage 3 T1DM (129). While the FDA-approved indication encompasses all people (8 years and older) with Stage 2 T1DM, it is not widely accessible globally, restricting its standard of care status. Nonetheless, where approved, it can be offered to people with Stage 2 T1DM (129). This may offer insights for the treatment of LADY.

### 6.2 Non-pharmacological treatments

#### 6.2.1 Lifestyle modifications

Consistent with T1DM and T2DM, lifestyle changes benefit LADY patients. Clinically, many LADY patients achieve glycemic targets early through lifestyle interventions (diet control combined with exercise). One LADY patient lost 6 kg after three months of a calorie-reduced diet and physical exercise, with glycated hemoglobin decreasing from 8.0% to 5.9% (normal) and blood glucose dropping from 7 mmol/L (fasting) to normal levels (fasting and postprandial) (9). Nevertheless, as the condition advances, lifestyle modifications alone become inadequate for disease management (**Table 2**).

**Table 2:** LADY-related case reports.

Year	Country	Age	Initial symptoms	Antibodies	Time of antibody appearance	Family history	Genotype	Time of insulin initiation	Reference
2000	Germany	8	Overweight, elevated blood glucose	ICA, GADA, IA-2A	At diagnosis	Both maternal grandfather and grandmother with T2DM	DRB1*0301/0401-DQB1*0201/0302	Still not needed 12 months after initial diagnosis	(9)
		8	Overweight, elevated blood glucose	ICA, GADA, IA-2A	3 months after initial diagnosis	Grandmother with T2DM	DR3/4, DQ*0201/0302	9 months after initial diagnosis	
2008	UK	15	Overweight, elevated blood glucose, glycosuria, moderate ketonuria	GADA, ICA	Not applicable	Sister with T1DM	Not applicable	4 years after initial diagnosis	(69)
2020	Japan	12	Normal weight, elevated blood glucose, diabetic	IA-2A	Initially negative, positive after 3 years	Uncle has T2DM	Not applicable	Not applicable	(70)
2024	UK	18	Overweight, elevated blood glucose, glycosuria, polyuria, irritable thirst and weight loss	GADA, ICA, IA-2A, ZnT8A	Not applicable	Not applicable	Not applicable	10 months after initial diagnosis	(11)

#### 6.2.2 Vitamin D supplementation

The involvement of vitamin D in the management of LADA has been acknowledged, but its value in LADY requires further investigation. Beyond its classical role in regulating calcium and phosphorus metabolism, vitamin D demonstrates potential immunomodulatory effects that may confer protection against autoimmune diabetes by influencing innate immune mechanisms. Vitamin D deficiency is frequently observed among individuals with LADA, and increased vitamin D exposure—achieved through supplementation and dietary intake—has been correlated with a decreased incidence of LADA (130). Though limited, case reports, case-control studies, and randomized clinical trials have investigated the effects of vitamin D supplementation on glucose regulation, residual β-cell function, and GAD65 antibody levels (131, 132). Notably, one study indicated that the administration of 1-alpha-hydroxyvitamin D3 [1α(OH)D3] in combination with insulin therapy resulted in improved fasting C-peptide concentrations in LADA patients compared to insulin treatment alone (133). Preliminary results suggest vitamin D supplementation may enhance glycemic control, preserve β-cell function, and reduce autoimmune activity. Given its widespread availability and favorable safety profile, vitamin D supplementation represents a viable adjunctive therapeutic strategy for patients with LADY.

## 7 Prospects and summary

LADY—a distinct subtype within the autoimmune diabetes spectrum—is increasingly recognized for its clinical importance. This review synthesizes current understanding of LADY, characterized by slowly progressive autoimmune β-cell destruction and significant heterogeneity, combining the autoimmune basis of classic T1DM with the initial clinical phenotype of T2DM. Notably, LADY prevalence among youth with suspected T2DM reaches approximately 10%. However, due to substantial overlap in early clinical presentation with T2DM, misdiagnosis rates remain high, posing a severe challenge for accurate identification.

Genetically, LADY resembles T1DM and harbors T2DM-associated variants. Immunologically, cell-mediated destruction predominates, with stronger autoimmunity than LADA. Diagnosis hinges on three pillars: youth onset, positive islet autoantibodies, and ≥ 6–12 months without insulin requirement. LADY treatment strategies remain controversial and guideline-deficient: while insulin is the ultimate therapy, the efficacy of early intervention in preserving residual β-cell function needs clarification. Oral hypoglycemic selection requires caution, and immunomodulatory therapies and vitamin D as adjuncts show potential.

In conclusion, LADY is a significant yet underrecognized autoimmune diabetes subtype. Key needs include standardized diagnostic criteria and age cutoffs, validated screening tools, large-scale genetic studies to identify LADY-specific loci, and mechanistic research to explain heightened autoimmunity. The ideal goal is optimal glycemic control with β-cell preservation. Due to marked heterogeneity, individualized therapy tailored to each patient’s phenotype may be best. LADY is an important and underappreciated form of diabetes in young individuals. Through multi-omics integration, multidisciplinary collaboration, and targeted interventions, earlier recognition and personalized treatment can be achieved, ultimately improving quality of life.
